# The Effects of Leg Length Discrepancy on Stability and Kinematics-Kinetics Deviations: A Systematic Review

**DOI:** 10.1155/2018/5156348

**Published:** 2018-07-11

**Authors:** Nurul Azira Azizan, Khairul Salleh Basaruddin, Ahmad Faizal Salleh

**Affiliations:** School of Mechatronic Engineering, Universiti Malaysia Perlis, Arau, Perlis, Malaysia

## Abstract

Various studies have examined body posture stability, including postural sway and associated biomechanical parameters, to assess the severity effects of leg length discrepancy (LLD). However, various viewpoints have been articulated on the results of these studies because of certain drawbacks in the comprehensive analysis of the effect of variations in LLD magnitude. Therefore, this systematic review was performed to help focus on the current findings to help identify which biomechanical parameters are most relevant, commonly used, and able to distinguish and/or have specific clinical relevance to the effect of variations in LLD magnitude during static (standing) and dynamic (walking) conditions. Several electronic databases containing studies from the year 1983 to 2016 (Scopus, ScienceDirect, PubMed, PMC, and ProQuest) were obtained in our literature search. The search process yielded 22 published articles that fulfilled our criteria. We found most of the published data that we analyzed to be inconsistent, and very little data was obtained on the correlation between LLD severity and changes in body posture stability during standing and walking. However, the results of the present review study are in line with previous observational studies, which describe asymmetry in the lower limbs corresponding to biomechanical parameters such as gait kinematics, kinetics, and other parameters described during static (standing) postural balance. In future investigations, we believe that it might be useful to use and exploit other balance-related factors that may potentially influence body posture stability.

## 1. Introduction

Leg length discrepancy (LLD) is also known as anisomelia, which is the term that is frequently used in the literature to describe the phenomenon of unequal lengths of the lower limbs [1]. LLD can be divided into two main subtypes—structural LLD, which is concerned with the shortening of bone structures, and functional LLD, which is defined as any mechanical changes that alter the posture of the lower extremities, for example, knee flexion. A report study from 2007 stated that LLD occurred almost in 70% of the general population [2]. Recently, researchers have found that only 1/1000 people who have LLD greater than 2 cm have noted changes in biomechanical gait [3, 4]. It has been observed that various common factors such as pain and arthritis may lead to spine, knee, and hip distress in patients and cause functional LLD [4].

It has been shown that biomechanical changes, such as those in plantar flexion and ankle posture, during standing and walking contribute to pelvic tilt in the coronal and sagittal planes [5, 6]. These findings cannot be extrapolated to all other relevant studies, of which only a few evaluated postural sway by assessing additional variable parameters such as center of pressure (COP), center of mass (COM), and other kinetics parameters [7–9].The authors feel that the analyses of COP, COM alone, and other parameters as stated above are likely to become more common in the future and this can affect balance and postural stability, which can be used as an indicator of static and dynamic control via postural sway. Recently, gait analysis has been used to evaluate biomechanical and physiological parameters during standing or walking with the use of a force platform device [10, 11]. Many evidence-based medical studies have been conducted to assess biomechanical changes that correspond to patient demographic characteristics and the environment. For example, patients with osteoarthritis and LLD after total hip arthroplasty (THA) may need to adapt to adjustment strategies to regain stability during locomotion [12–14]. Recently, many studies have been conducted to assess the correlation between biomechanical parameters and environmental factors in cases of LLD. To date though, how does LLD affect the kinematics and kinetics of gait and how does LLD affect standing still balance [14] and walking [4] remain unclear. Among the different methods for assessing balance and postural stability, the different types of LLD could be affected differently by the grade of LLD. Hence, the present systematic review provides a summary of the literature on body posture stability and considers the different outcomes and relevant biomechanical parameters such as kinematics and kinetics affected by the different degrees of LLD.

## 2. Methods

### 2.1. Literature Search

Peer-reviewed journal articles were obtained by searching electronic databases such as Scopus (1983–2016), ScienceDirect (2002–2016), PubMed (2001–2016), PMC (1999–2016), Medline (2010–2016), and ProQuest (2000–2016). The key words “leg length discrepancy,” “postural stability,” and “postural sway” were used as search terms to find studies relating to the effects of structural LLD on postural body stability. A Medical Subject Headings search was used to broaden the search using combinations of keywords which included “kinematic,” “kinetic,” and “gait analysis.” All online biomechanical papers were retrieved and screened to ensure that the results of the database search were relevant and related to other articles. The final finding was done to restrict our findings to the scope of the articles. The relevant articles that were chosen were based on the scope of the study for any human abnormalities in lower extremities. Additional citation/articles are also included to support the argument and case from the selected articles.

### 2.2. Inclusion/Exclusion Criteria

The literature search only included articles written in English. For convenience, we only included studies with experimental protocols that used a quantified measure of body postural control and performed gait analysis using laboratory devices such as force platforms or other specialized equipment. It is beyond the scope of the present study to examine simulation software modeling; however, studies were considered if they used human participants with different medical backgrounds and if their illness or comorbidities did not affect LLD or posture balancing (in standing and walking). In contrast, patients with amputated limbs or neuromuscular disorders were excluded. Studies that assessed gait analysis using prosthetic designs, shoe soles, and wooden blocks were included. No limitations were applied with regard to age, sex, BMI, and time of LLD occurrence of the participants. Articles from the same author were thoroughly checked to avoid any duplicate articles.

### 2.3. Quality Controls

Screening of the search results was performed by two authors (Nurul Azira Azizan, Khairul Salleh Basaruddin) per inclusion criteria. Following screening, final articles were retrieved and separated accordingly from the duplicated articles in different databases. Titles and abstracts were read thoroughly, and those that met the following criteria were included: (1) human participants, (2) focus on asymmetry of limbs, (3) measured posture balance stability, and (4) reviewed study design of experimental protocol.

### 2.4. Assessment of Research Quality

The assessment of articles used a systematic quality method for review and analysis, which helped assessing the quality of retrieved articles and the most relevant information from those articles. Aside from those in the current review described here, there are no standardized methods to assess the credibility of each of the reviewed paper. To assess credibility, 14 questions were adopted from Ku et al. [15]. Each question was evaluated as “2” if the answer fulfilling the standard questions was “yes” and “1” if it was limited in detailed information. Invalid questions were given “0” or “no,” and not applicable questions were given “NA.” It should be noted here that the adequacy of the biomechanical LLD evaluation of standing and walking for balance stability was resolved through discussion by each author.

## 3. Results

### 3.1. Primary Search Results

**Figure 1:**
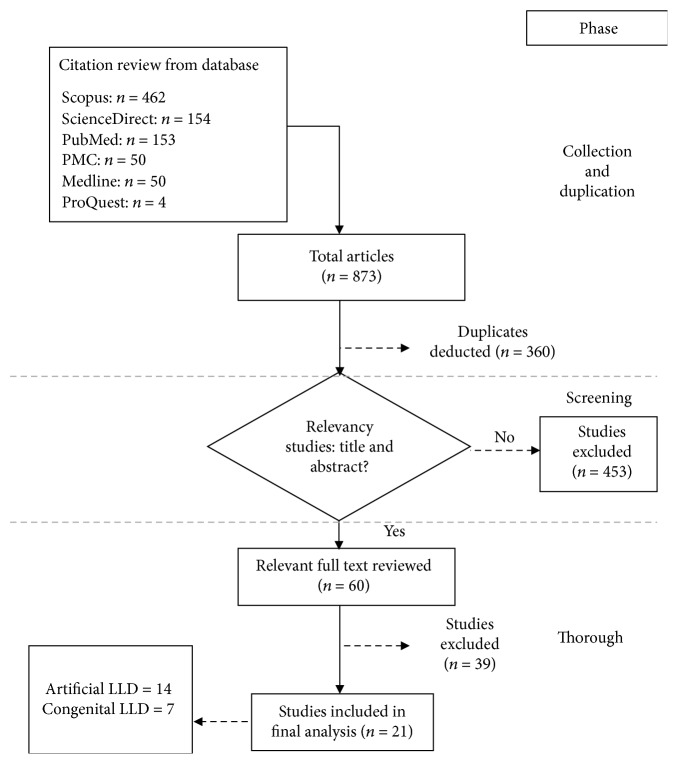
Flowchart procedure showing the study selection process from reviewed papers.

The findings were limited in terms of quantity of information, and the authors then performed full-text reviews of articles. Twenty-two retrieved articles were finalized after a thorough screening process was performed.  Figure 1 shows the present study's systematic review process. The database screening process yielded a total of 873 articles. However, 360 of these articles were identified as duplicates and removed. Titles and abstracts were reviewed for the relevance of the studies performed, after which 453 articles were further excluded. Additional screening was executed by reading the rest of the articles in totality to determine the aims of studies based on the standard parameters that was evaluated. This yielded 22 articles that considered standing and walking posture for LLD cases after eliminating further 40 articles.

### 3.2. Analyzed Data Quality

Quality ratings of the 22 reviewed papers are listed in Table 1. The quality score of the reviewed articles ranged from 76.92% to 96.43%. Most of the articles provided complete information regarding their objectives, study design, main outcomes, and conclusions based on the 14 questions we used. Other factors that could have contributed to the understanding of the question were not used in this study.

### 3.3. Participant Characteristics

Table 2 presents a list of the physical characteristics and anthropometric parameters from the 22 articles that we reviewed comprehensively. Seven articles compared a healthy group (control) and LLD group [3–5, 7–11, 13, 16, 17]. There was a notable paucity of well-controlled studies investigating participants in groups that had various disorders: (1) after total hip arthroplasty, (2) knee osteoarthritis, (3) lower back pain [24], and (4) acromegaly [16–19]. Most of the participants were adults, young adults, average-age adults, and older adults, whereas very few published studies focused on children with Legg–Calvé–Perthes disease (LCPD) [16, 19]. Several articles provided complete information on patient characteristics. Seven of the studies provided insufficient details regarding gender [2, 4, 5, 7, 10, 18] and anthropometric parameters [19]. The highest number of participants seen studied in an experiment was 60, whereas the lowest was two.

### 3.4. Experimental Study Procedures

There are two possible approaches that have been used for the investigation of LLD, where nine articles studied on congenital LLD [2, 12, 16, 19–24] and 13 articles studied on induced artificial LLD [3–5, 7–11, 13, 14, 17, 18] as listed in Table 3. Most of the studies performed their experiments during walking. However, 11 articles also conducted experiments during standing. Most cases of artificially induced LLD were created by providing participants with insoles of differing thickness, with the exception of one study in which a gait walker was used [11]. Twelve studies performed three to five trials when measuring walking performance or balance and posture while standing. Meanwhile, nine of the studies did not clearly provide the number of trials used. Only Zhang et al. [11], Resende et al. [3, 18], and Maeda et al. [9] had clearly stated the number of trials performed for each condition in their experiments.

Previous studies have tested the efficacy of balance performance by using various force platforms including the Vicon Systems [9, 11, 20], Musgrave™ pedobarographs [10, 13, 14], footprint pressure plate systems [13, 14], zebris [5], MatScan and T-Scan II computerized occlusal analysis systems [9], scanograms [20], Berg Balance Scale [22], and photogrammetry and mechanical electric elevation treatment tables [23]. In some studies, static posture was measured using tape measurements [5, 19, 21], goniometers [16], video recording [20], palpation meter inclinometers, and bubble inclinometers [21]. From ten articles using reflective markers [2, 3, 7, 8, 11, 12, 19, 20, 23], only two mentioned the total number of reflective markers used [11, 12]; however, five articles mentioned the anatomical landmarks used for marker placement, such as the tibial tubercles (ankle and subtalar joints at the foot) [2]; pelvis, thighs, shanks, and feet (anterior and posterior regions for multisegment recordings) [3, 20]; thigh, foot, pelvis, anterior-posterior (AP) iliac spines and iliac crest, greater lateral trochanter, medial-lateral (ML) epicondyles and malleoli, head of first and fifth metatarsals at the feet, and ankle [11]; and medial malleolus, medial femoral condyle and major trochanter for hips, and knees and ankles [16]. In addition, the insole material used to induce artificial LLD also has a significant influence on the changes in kinematics parameters, for instance, ankle eversion and hip abduction [11]. There are several materials that are commonly being used (flexible polyurethane, polypropylene, wooden boards, high-density ethylene vinyl acetate, pelite, hard cork, leather nylon mesh tissue, plastazote ethylene vinyl acetate and poron, thermoplastic alloy (TPA), and foot mask).

### 3.5. Kinematics and Kinetics Responses

Table 4 lists the summary of the kinematics and kinetics responses for congenital or artificial LLD with respect to posture stability. It provides outcome measurements from gait analysis of kinematics parameters [2–5, 8, 10, 11, 15, 20, 23] and kinetics parameters [9, 14, 19]. All studies used different LLD degrees during standing or walking with a view of multiple planes. Recently, only six articles used sagittal and frontal planes to examine the postural effects of LLD, whereas only one article analyzed all planes [4]. It can be seen in Table 4 that the majority of directions used were the AP, ML, and bilateral (BL). Several studies stated that the participants were selected based on a degree of homogeneity at several different levels of LLD between 0 and 5 cm. There were 24 kinematics parameters (as listed in Table 4) that were used in LLD analysis, including ankle eversion/dorsiflexion angle; hip and knee adduction angle; hip extension angle; knee flexion angle; pelvic obliquity angle; foot dorsiflexion; plantar flexion and inversion angle; trunk and pelvic external-internal rotation; trunk forward tilt; pelvis lateral tilt; range of motion for the ankle, knee, and hip; mean peak plantar pressure; stance contact duration; contact area; maximum foot pronation and supination; head horizontal alignment; and anterior-superior iliac spine horizontal alignment. Fourteen kinetics parameters were found in the reviewed articles, including weight distribution (WD); ankle inversion/dorsiflexion/flexion/plantar flexion moment; knee flexion/adduction moment; hip flexion/adduction/extension moment; power for ankle, knee, and hip; and vertical ground reaction force (VGRF). Based on the reviewed articles, the statistical significance was analyzed using SPSS software [11, 18] including *t*-tests [6, 11, 13], Kruskal–Wallis tests, chi-squared tests [12], Kalmogorov–Smirnov tests, Shapiro–Wilk tests [3, 15, 20, 23], Wilcoxon signed-rank tests [8], Mann–Whitney *U* tests [17], one-way repeated measures analysis (ANOVA) [5, 23], two-way multivariate ANOVA, Friedman two-way ANOVA, and Dunn's multiple comparisons [9, 21] as deemed appropriate for each study. The influence of sample size on the magnitude of the effect for group differences was also analyzed by the two articles [16, 20] using Cohen's *d*.

### 3.6. Other Parameters

Table 5 summarizes the parameters used to measure postural balance control including mean center of pressure (COP), COP path length, COP total trajectory length, COP area, and center of mass (COM). These parameters were separated due to the specific objectives focused by the researcher based on their scope of study. Intervention on this information is important to acquire direct knowledge of the parameters used to describe postural stability. Only three articles provided the sample rate and length of experiments using a force platform over several changes in LLD degree [6, 9, 24]. The parameters stated above contributed to an asymmetrical gait while standing or walking in each gait cycle, and posture conditions are as follows: (1) heel strike to toe-off and stance [11]; (2) heel to toe from midline of the right foot to the left foot along the AP and ML axes [6]; (3) AP and ML during eyes-open and eyes-closed trials [19]; (4) AP and ML [5, 10, 15]; (5) natural standing posture-centric occlusion, right heel lift-centric occlusion, left heel lift-centric occlusion; and (6) feet apart during eyes-open and eyes-closed trials [9].

## 4. Discussion

### 4.1. Quality of Search

The purpose of this systematic review was to examine reliable biomechanical parameters that are commonly used to distinguish and/or have specific clinical relevance to the effect of variations in LLD magnitude during static (standing) and dynamic (walking) conditions. Comparing parameters used in each study is crucial in understanding how to improve postural control in cases of LLD. Based on previous research, the consideration of these two related cases, that is, congenital and artificially induced LLD, should not be discussed in separate tables, since both cases are involve in the findings in Tables 3, 4, and 5. In the present study, 22 articles were included, whereby 14 of them were further analyzed for their kinematics and kinetics parameters related to LLD. The 8 remaining articles were analyzed and summarized based on the postural sway parameters; however, Walsh et al. [4] and Murrell et al. [19] described four basic kinds of validity: logical, content, criterion, and construct of their paper in order to refer and select papers in this study. Specifically, no meta-analysis was performed for the quality assessment of the methods, whereas there were shortcomings with the interpretation that were affected by the tasks performed, task duration, task difficulty, and sample rate of data collection. Standardized protocols would systematically improve the results of such studies to avoid any uncertainty and bias in the results.

In the reviewed studies, additional discussion can be divided into participant's characteristics and demographics, kinematics, and kinetics responses. Subsequently, evaluation of static (standing) activity of postural control is discussed since it is the factor that most studies in LLD are involved with. Participant characteristics were heterogeneous and there was a tendency to categorize patients into groups based on sex (female or male), number of participants, and anthropometric parameters (age, height, weight, and BMI). Lopes et al. [22] observed no significant differences between healthy participants and patients with anisomelia regarding age, weight, height, or BMI, whereas D'Amico et al. [24] observed that the participants' heights need to be taken into account, which was subsequently done by several authors [1, 2, 4, 11]. This is because taller participants show slightly higher LLD. Several authors [3, 5, 10, 11, 22, 23] attempted to draw fine distinctions between mild (difference < 3 cm), moderate (between 3 cm and 6 cm), and severe (>6 cm) LLD, while others questioned the usefulness of different categories because participants in different clinical and experimental LLD studies varied in gender, age, and weight [17]. Regarding the category or conditions in the group of studies, Lopes et al. [22] reported no significant differences between the control and acromegaly groups. This is contradicted by Wünnemann et al. [8], who only studied healthy participants and neglected true patients since their gait could be disturbed by other factors such as pain and neuromuscular dysfunction influencing motor control. Although differences of opinion on the types of LLD still exist, three articles investigating control groups (healthy participants) and experimental groups (LLD symptoms) [2, 15, 24] found that true LLD leads to less stability than healthy controls showed. Only Ali et al. [2] demonstrated that changing the foot position by 1 cm affected the foot posture by changing it from pronation to supination. Only a few studies available in the literature investigated the correlation between LLD type and body posture stability.

### 4.2. Kinematics and Kinetics Responses

A few published studies have provided quantitative evidence of the changes in biomechanical parameters corresponding to the degree of LLD. Several studies have tested the efficacy of kinematics and kinetics parameters during standing, walking, or both. Additional parameters such as step length, step duration, stride length, stride duration, single leg support, and double leg support for gait analysis appear to have an influence on behavioral aspects of LLD types with respect to the different changes that occur during gait. It has been shown that there are changes in balance adjustment when the WD was concentrated more in the shorter limb than the longer limb. However, Swaminathan et al. [14] found a significant difference when the height of the right leg was raised. This finding is in agreement with Maeda et al. [9] and Aiona et al. Reference [20] is shown significant difference when the height of the right leg was raised. This result is somewhat counterintuitive, considering that no significant difference in right and left leg control is seen with 0.1 cm change in height in the coronal and sagittal planes. However, according to the degree of LLD height, WD has a significant influence when the LLD is increased to 6 cm or more in the coronal plane or 4 cm or more in the sagittal plane [9]. The study by [9] approach showed little evidence for a correlation between kinematics parameter and posture stability. The finding regarding WD is helpful in providing insight to orthopedists or physiotherapists for limb lengthening to prevent future rehabilitation needs.

However, from the viewpoint of kinematics responses, Resende et al. [3] found that the shorter limb demonstrated increased ankle plantar flexion angles during the loading response phase in the sagittal plane [18]. This change is also supported by other articles. Stief et al. [16] reported significant differences in ankle dorsiflexion moments, and the shorter limb was affected while increasing pelvic obliquity. This effect was only found for changes in the sagittal plane. Walsh et al. [4] reported that pelvic obliquity in the sagittal plane showed significant changes when LLD was induced artificially at 2.5 cm, which is also supported by Stief et al. [16] and Resende et al. [3]. These findings occurred in both static standing and walking conditions. Furthermore, kinematics parameters accommodate larger discrepancies by altering movement in the coronal plane, which was observed by Resende et al. [3] and Wünnemann et al. [8]. Parameters that also play an important role are the range of motion at the ankle, knee, and hip during stance phase [11, 16, 25] in the sagittal and coronal planes. Zhang et al. [11] tried to determine the effect of the VGRF response by comparing the patterns between raising the heel in the longer leg and shorter leg (as a control) during terminal stance phase, which resulted in dissimilar patterns, because the loading response showed a higher impact on the longer leg. Park et al. [5], Wünnemann et al. [8], and Maeda et al. [9] induced artificial LLD between the left and right legs as a control. Additional parameters that may be relevant for LLD analysis, including step length, step duration, and single leg support, support the assertion that no significant changes are seen from 0 cm to 1 cm but there are changes from 2 cm to 3 cm. The most surprising finding is that the stride length and stride duration showed no significant differences during walking.

Further, Faraj et al. [10] studied mean peak plantar pressure, stance contact duration, and contact area, which showed significant changes in the coronal plane at ±2.25 cm LLD. A broader perspective was adopted by Seeley et al. [17], who argued that LLD between 1.0 cm and 2.3 cm exhibited significantly less symmetrical gait in the ankle and knee joint moments compared to LLD that is less than 1 cm. This finding was contradicted by Murrell et al. [19], who found very little differences between LLD degrees (≥1 cm and <1 cm) for several of the dependent kinetics variables. Moreover, kinetics parameters such as power and moment have been carried out on each direction and position (flexion, abduction, and extension) for the ankle, hip, and knee. Various studies indicated that ankle dorsiflexion, hip flexion, pelvic anteversion, and pelvic retroversion occurred during mild LLD in the frontal plane [3, 16]. No significant differences were seen in the sagittal plane during walking [11], while another author demonstrated that changes in the sagittal plane created small but significant changes [5]. The plane in which movement was viewed relates to the method used, for example, in radiography (CT scan, X-ray, and scanogram) [5, 9, 19, 22, 23]. However, Zhang et al. [11] were much more concerned with the materials used for sole inserts or the method as raised heel heights lead to decreases in knee and hip abductors based on the joint kinetics and to increases in the knee extensors. But this was contradicted by Mahar et al. [6] who argued that increased LLD results in no increase in postural sway. Overall, the evidence demonstrates greater need to assure the effects of leg length discrepancy for body stability in terms of the participant's confidence level (fear of fall) and trial testing for each experimental condition.

### 4.3. Evaluation in Static (Standing) Balance

Recent studies on LLD deal with the effects of LLD on the body's stability and posture. It has been documented that ML position of COP 1 cm towards the longer limb increased the magnitude of postural sway in a study done by Ki et al. [5], who pointed out that any increase in LLD led to an increase in COP path length. Furthermore, Maeda et al. [9] argued that COP (total trajectory and area) did not affect postural stability with a heel lift (<1 cm) under either foot. Several COP parameters (mean, total length trajectory, path length, and locus contact area) showed significant changes in the study by Ki et al. [5]. COP path length was significantly longer when LLD was at least 3 cm (mild LLD), and this clearly shows changes in body stability caused by LLD during standing; this was also demonstrated by Maeda et al. [9]. Mahar et al. [6] performed a similar series of experiments in the 1960s to show that mean COP position affects postural sway during walking. They demonstrated that 1 cm LLD caused changes in the ML direction. Recent studies on LLD deal with the question of the effect of LLD on body stability and posture.

Although research has been conducted into the effects of dynamic WD on static function, there are no articles examining the dynamic function. High BMI will show larger variations in biomechanical parameters; however, there are no studies to show the correlation between biomechanical parameters and patients' characteristics, such as anthropometric parameters (age, height, weight, and BMI) [4, 11, 22, 26]. The interactions and contributions of these factors cannot be found in the literature search done by us/our team. Although early findings were only limited to different sexes, there were no studies found with participants of the same sex with different pathological disorders. However, all the previously mentioned methods suffer from some limitations in this review, such as the fact that the search strategy only used English databases. The reporting criteria were not necessarily limited to relevant standard methods in the present study.

## 5. Conclusions

The present review shows 22 most common articles with specific parameters published until now. In this study, we noticed that LLD patients are often better predictors of imbalance compared to the normal population due to the changes that occur to their posture and physical shortcomings. This review focused on kinematics, kinetics, and other variable parameters that are associated with the effects of LLD on body stability. Each of the parameters was associated with the effects of height, confidence levels, and age effects, which correlated with the elevation of LLD. However, previous researchers [3, 5, 17] have noted that there are no specific parameters to describe postural stability due to inconsistencies found in relevant studies that have been done. No concrete data was found on correlations between height and weight with the other parameters mentioned, though they certainly contributed to changes in body posture and stability in cases of LLD. Therefore, investigation into the influence of the body height and weight ratio with respect to the degree of artificial LLD is recommended to be carried out in future studies.

## Figures and Tables

**Table 1:** Overall rating score from reviewed papers.

Authors	Questions															
	1	2	3	4	5	6	7	8	9	10	11	12	13	14	Overall score	Overall %
Stief et al. [16]	2	2	1	0	NA	NA	2	2	2	2	2	2	NA	2	19/20	95.00
Seeley et al. [17]	2	2	2	NA	1	2	2	2	2	2	2	2	2	2	25/26	96.15
Ali et al. [2]	2	2	2	2	2	2	2	2	2	2	2	2	1	2	27/28	96.43
Swaminathan et al. [14]	2	2	1	NA	2	NA	2	2	2	1	2	2	1	2	21/24	87.50
Renkawitz et al. [12]	2	1	1	NA	2	1	2	2	2	2	2	2	1	2	22/26	84.62
Resende et al. [3]	2	2	2	1	2	2	2	2	2	2	2	2	2	NA	25/26	96.15
Walsh et al. [4]	2	2	2	1	2	2	2	2	2	2	2	2	2	2	27/28	96.43
Mahar et al. [6]	2	2	1	1	2	2	2	2	2	2	2	2	1	1	24/28	85.71
Resende et al. [18]	2	2	2	1	1	2	1	1	1	0	2	2	2	2	21/26	80.77
Murrell et al. [19]	2	2	2	1	2	2	2	2	2	2	2	2	2	2	27/28	96.43
Roerdink et al. [7]	2	2	1	1	2	2	1	2	2	2	1	2	1	1	22/28	78.57
Wünnemann et al. [8]	2	2	1	2	1	2	1	1	2	2	2	2	2	2	24/28	85.71
Maeda et al. [9]	2	2	2	0	2	2	2	2	1	2	2	2	1	2	24/26	92.31
Aiona et al. [20]	2	2	2	1	2	2	1	2	1	1	2	1	1	1	22/28	78.57
Faraj et al. [10]	2	2	2	2	2	1	1	2	1	2	2	2	0	1	23/26	88.46
Zhang et al. [11]	2	1	2	2	2	2	2	2	1	1	1	1	NA	1	20/26	76.92
Krawiec et al. [21]	2	2	2	2	2	2	0	2	1	2	2	2	NA	2	23/24	95.83
O'Toole et al. [13]	2	2	2	0	2	2	2	2	1	1	2	2	1	1	22/26	84.62
Lopes et al. [22]	2	2	2	2	2	2	1	1	1	0	2	2	NA	1	20/24	83.33
Schneider et al. [23]	2	2	1	NA	2	2	2	2	1	2	2	2	1	1	22/26	84.62
Park et al. [5]	2	2	2	2	2	2	2	2	2	2	2	2	1	1	20/24	92.86
D'Amico et al. [24]																
																

Significance evaluation: 2: yes; 1: limited detail; 0: no; NA: not applicable.

**Table 2:** Participants' characteristic.

Author	Condition/category	Number of participants	Gender		Anthropometric parameters (mean/SD)
			Male	Female	
Ki et al. [5]	Healthy adults	17	NM	NM	Age (years): 25.76 ± 1.76
					Height (cm): 167.12 ± 7.39
					Mass (kg): 64.94 ± 10.57

Swaminathan et al. [14]	Healthy	20	11	9	Age (years): 19–60 ± 38.85
					Height (cm): 152–190 ± 172.76
					Mass (kg): 50.8–90.0 ± 71.14

Renkawitz et al. [12]	After THA	60	29	31	Age (years): 61.2 ± 7.2
					Height (cm): 167.12 ± 7.39
					BMI (kg/m^2^): 26.5 ± 3.8

Resende et al. [3]	Healthy	19	10	9	Age (years): 25 ± 6.0
					Height (cm): 174 ± 7.2
					Mass (kg): 72.3 ± 11.5

Walsh et al. [4]	Healthy	7	NM	NM	NM

Mahar et al. [6]	Healthy	14	8	6	Age (years): 28.3 ± 1.0
					Height (cm): 168.7 ± 8.6
					Mass (kg): 63.4 ± 10

Resende et al. [18]	Knee OA	15	6	9	Age (years): 67 ± 8.8
					Height (cm): 169 ± 0.07
					Mass (kg): 88.9 ± 20.1

Murrell et al. [19]	Healthy (control group)	11	3	8	NM
	LLD (experiment group)	9	4	5	NM

Seeley et al. [17]	LLD (<1 cm)	19	11	8	Age (years): 30 ± 6.0
					Height (cm): 174 ± 0
					Mass (kg): 73.9 ± 5.7
	LLD (≥1 cm)	7	5	2	Age (years): 28 ± 8.0
					Height (cm): 176 ± 7.0
					Mass (kg): 74.6 ± 16.2

Roerdink et al. [7]	Healthy	15	15	NM	Age (years): 22 ± 1.0
					Height (cm): 178 ± 5.0
					Mass (kg): 77 ± 5.0

Wünnemann et al. [8]	Healthy	15			Age (years): 26.1 ± 9.0
					Height (cm): 180.3 ± 6.6
					Mass (kg): 75.7 ± 7.9
			11		Age (years): 26.5 ± 10.6
					Height (cm): 183 ± 5.4
					Mass (kg): 79.4 ± 5.2
				4	Age (years): 24.8 ± 1.7
					Height (cm): 173 ± 2.9
					Mass (kg): 65.5 ± 2.9

Stief et al. [16]	Healthy	15	11	4	Age (years): 7.0 ± 2.6
	LCPD children	12	10	2	

Maeda et al. [9]	Healthy	30	15	15	Age (years): 19–33 ± 25.6
Aiona et al. [20]	LLD children (≥2 cm)	45	21	24	Age (years): 5.7–20.6 ± 3.5
	LCPD	4			
	Hip dysplasia	9			
	Growth plate damage	6			
	Congenital short femur	5			
	Congenital short tibia	7			
	Others	9			

Faraj et al. [10]	Healthy	1	2	NM	Age (years): 22
					Height (cm): 179
					Mass (kg): 68
	LLD (2 cm)	1			Age (years): 44
					Height (cm): 174
					Mass (kg): 65

Zhang et al. [11]	Healthy	11			Age (years): 27.4 ± 7.8
					Height (cm): 176 ± 8
					Mass (kg): 72.0 ± 13.4
			6		Age (years): 29.7 ± 9.9
					Height (cm): 182 ± 5.0
					Mass (kg): 80.9 ± 9.3
				5	Age (years): 24.6 ± 3.4
					Height (cm): 169 ± 3.0
					Mass (kg): 61.3 ± 9.0

Ali et al. [2]	Healthy (experimental group)	5	NM	NM	NM
	Patients with flexible flat feet (clinical group)	3			

Krawiec et al. [21]	Healthy	44	24		Age (years): 19.7 ± 1.2
					Height (cm): 185.1 ± 7.3
					Mass (kg): 81.4 ± 9.9
				20	Age (years): 19.4 ± 12
					Height (cm): 168.6 ± 6.9
					Mass (kg): 64.2 ± 6.2

O'Toole et al. [13]	Healthy	15	7	8	Age (years): 17–37 ± 26.5
					Height (cm): 154–193 ± 167
					Mass (kg): 55–90 ± 66.5

Lopes et al. [22]	Acromegaly	28	17	11	Age (years): 48.6 ± 12.5
					Height (cm): 165 ± 0.11
					Mass (kg): 79.2 ± 15.0
					BMI (kg/m^2^): 28.8 ± 3.37

Schneider et al. [23]	LBP	45	NM	NM	Age (years): 18–65 ± 41.5

D'Amico et al. [24]	LBP	94	NM	NM	Age (years): 46.3 ± 16

NM: not mentioned; THA: total hip arthroplasty; LLD: leg length discrepancy; LBP: lower back pain; LCPD: Legg–Calvé–Perthes disease; SD: standard deviation; OA: osteoarthritis

**Table 3:** Data extraction from reviewed articles.

Study	Protocol	Instrumentation	Sampling rate (Hz)	Material of artificial LLD	Outcome measures	Findings
Zhang et al. [11]	Walk in 5 different levels of walking trials. Each trial has 3 conditions: (i) 2 testing boots and (ii) 1 pair of laboratory shoes.	Force platform 6-camera motion analysis system Reflective and tracking markers Gait walker and equalizer Laboratory shoes	600 Hz	Polyurethane outsole Polypropylene midsole Hard foam as the insole	GRF impact, ROM, and COM	Different heights of insole and material show initial impact from GRF peak. ROM is less significant during eversion and hip adduction for walking trials using gait walker. Kinematics frontal plane shows greater significance than sagittal plane.
Swaminathan et al. [14]	Stand still on the pedobarograph with both feet and a 2 s recording was taken to establish their body weight.	Pedobarograph Musgrave Footprint pressure plate system	56 Hz	Wooden boards	WD	WD is significant at the shorter limb than the longer limb.
Renkawitz et al. [12]	Walk on a predetermined 10 m walkway with a self-selected number of 3- or 5-time trials.	3D (CT) scans 3D motion gait analysis of the lower extremity 6 digital cameras with a video 27 retroreflective markers	70 Hz	NM	Hip ROM	LL and OS restoration within 5 mm > control normalized walking LL/OS discrepancies > THA Healthy young = 45° Fit and healthy elderly = 35° Good postoperative gait = between 35° and 45°
Resende et al. [3]	Walk under 3 different conditions, wearing a combination of flat sandals with 6 trials: (1) Control: thick sandals bilaterally (2) Short limb: a thick sandal on the left foot and a thin sandal on the right foot (3) Long limb: a thin sandal on the left foot and a thick sandal on the right foot. Then, walk at self-selected speed.	12-camera motion capture system (Oqus 4, Qualisys), 6 force platforms (custom BP model, AMTI), marker, a pair of flat sandals (made of high-density ethylene vinyl acetate and were attached to the feet with Velcro™ (TM) straps)	1000 Hz	Sandals with high-density EVA	Fear foot dorsiflexion and inversion Ankle dorsiflexion Ankle inversion moments Knee flexion angle Knee flexion moment Knee adduction moment, Hip flexion angle Hip flexion moment Angle moment Pelvic ipsilateral	Kinematics mild LLD is of greater significance than non-LLD.
Walsh et al. [4]	Stand in front of the CODA system. A static analysis of their relaxed standing posture was obtained (the static test). Gait analysis Then, walk at self-selected speed and walking velocity.	3-D gait analysis using a CODA MPX 30® analyzer Sole of one foot using pelite.	NM	Sole of pelite	Static standing tests: Pelvic obliquity of the long limb Knee flexion of the long limb Dynamic walking test: Pelvic obliquity Hip, knee, and ankle flexion Swing dynamic walking test: Hip, knee, and ankle flexion	Pelvic obliquity occurred between 2 cm and 3 cm of LLD. Significant changes during knee flexion occurred at 2 cm. Significant changes in kinematics for the stance phase of both legs during walking
Mahar et al. [6]	Stand comfortably on a force platform with the knees extended and shoes removed and look ahead at a fixed object in a well-lit, quiet room. The foot angle was 12^″^ from the sagittal plane, and the base width at the heels was 10 cm.	Force platform	100 Hz	Insole lift cork	Mean COP	ML position of the COP toward the longer leg shows changes occurring at 1 cm. LLD raise resulted to no increase in the shift towards the longer leg. Changes increase in ML. Magnitude of postural sway
Resende et al. [18]	Walking under 2 different conditions: (1) Control: wearing flat thick sandals on both limbs (2) Short limb: wearing flat thin sandal on the OA limb knee and a flat thick sandal on the contralateral limb. Subject walked at self-selected speed, performing 5 trials per condition along a 15 m distance.	12-camera motion capture system 6 force platform AMTI Forefoot and rearfoot markers Tracking markers	200 Hz	Sandals with high-density EVA attached with Velcro	Kinematics: Rearfoot dorsiflexion-plantar flexion inversion-eversion Knee and hip flexion-extension, adduction, and abduction Trunk flexion-extension Kinetics: Ankle, knee, and hip internal moments in the sagittal and frontal planes	Biomechanics of mild LLD affect the kinetics chain with moderate knee OA during stance phase. Shorter limb increased pelvic and trunk external rotation stance. Mild LLD with knee OA caused lower back pain. Longer limb: Rearfoot plantar flexion angle increased. Ankle plantar flexion moment increased. Reduced hip abduction angle.
Murrell et al. [19]	Control group for minor LLD (±0.22 cm) Experimental group for 1/4 LLD which is ±0.11 cm Stand with barefoot on the force platform.	Force platform	10 Hz	NM	COP	No significant difference between the control group and experiment group Having LLD has significant changes than not having LLD in body stability.
Seeley et al. [17]	Stand still in front of absorptiometry scan. Then, the subject needs to perform standard gait analysis.	Body dual energy Absorptiometry scan Ruler 6 high-speed video cameras (motion analysis) 2 force platforms Reflective markers	60 Hz	NM	Joint moments and joint powers in the hip, knee, and ankle	Large LLD (between 1.0 cm and 2.3 cm) shows greater significant changes than small LLD (<1 cm) for each parameter.
Roerdink et al. [7]	Walk on a force platform with 5 trials along a 10 m walkway.	Total body dual energy Absorptiometry scans (DXA; Lunar DPX-IQ, Lunar Inc., Madison, WI, USA) Ruler 6 high-speed video cameras 2 force platforms Reflective markers	60 Hz	NM	BS plane joint angles, net joint moments, and joint powers in the hip, knee, and ankle	Gait symmetry has significant changes in LLD.
Wünnemann et al. [8]	The right foot with either the WCSTM or the Wedge Shoe™ The left foot with either the TwinShoe or the normal shoe For each shoe condition, 6 were made trials—3 right steps and 3 left steps on the force plate were recorded.	3 types of shoes: Wound care shoe system (WCSTM) OrthoWedge Healing Shoe Twin shoes 6-camera motion analysis 2 force platforms Reflective markers	60 Hz	Surface material: Leather nylon mesh tissue Insole material: Plastazote, EVA, and poron Sole material: TPA	Kinetic: Joint angles, GRF, joint moments, step length, and single-support time as the average values	Elevated shoe/sole on the CL foot led to gait alterations. Significant difference at the frontal plane Elevation leg shows greater hip flexion. Lower side showed elongated hip extension. Therapeutic shoes have greater significant changes at the movement patterns and load in the lower extremity and lower back. Shoes with elevated soles on CL have greater significant changes in alteration of gait kinematics for LLD's patience.
Park et al. [5]	Measure subject's leg length by using TMM. Artificial LLD was induced by using insoles at the left leg. Each subject would stand still in front of the radiographic device CTTT Then, measured using zebris FDM	Radiographic device CTTT zebris FDM Tape measurement (TMM) Insoles	NM	Insole	WD Mean COP path length Cobb's angle Gait parameter: Step length, step time, stride length, stride time, percentage of time in single-leg support, and percentage of time in double-leg support	Larger LLD (3 cm) increased COP path length and Cobb's angle. LLD (2 cm) has a significant difference in step length on the left (long) side, step time on the left (long) and right (short) sides, and single-leg support time on the left (long) side. LLD (1 cm) was significantly changed on the single leg support time for the short side.
Stief et al. [16]	Subject needs to be diagnosed by using goniometer-based clinical test protocol. All subjects were thoroughly familiarized with the gait analysis protocol.	Goniometer-based clinical test protocol 3D gait analysis T10 VICON motion capture system 8 infrared cameras 2 AMTI force plates Reflective markers	200 Hz	Insole	Flexion/ extension ROM for the knee and hip	LLD has greater significant changes than shortening LCPD Max. passive hip ROM has less significant changes than Max. passive hip abduction
Maeda et al. [9]	3 conditions of protocols: NS-CO: natural standing posture without a heel lift. RHL-CO: natural standing posture with a heel lift under the right foot. LHL-CO: natural standing posture with a heel lift under the left foot.	The MatScan system T-Scan II computerized occlusal analysis system	80 Hz	Hard cork	The total trajectory length of the COP/COF area LWD, AWD, and occlusion force distribution	Body posture: No significant difference between total trajectory lengths of COP, COP area, LWD, and AWD. Artificial LLD has greater significant changes than the control group affected by LWD. Natural standing posture: Heel LL: no significant difference in total trajectory length of COP, COP area, LWD, and AWD Artificial LLD has greater significant changes than control and it affected lateral foot pressure.
Aiona et al. [20]	Walk at self-selected speed along a 2 m walkway. Videotaped at the frontal and sagittal planes of each child	VICON clinical manager Videotape for frontal and sagittal 2 AMTI force plates 8 cameras 13 reflective markers Scanogram	60 Hz	NM	Compensation based on absolute LLD: Pelvis and knee flexion WD for the hip, knee, and ankle	Short femur: WD at the ankle has greater significant changes than the control group. Joint parameters have greater significant changes than the control group. Short tibias: WD has greater significant changes than the control group. Hip disorder has greater significant changes than normal hip. Joint parameters have greater significant changes than the control group.
Faraj et al. [10]	Walk with 3 multisteps walking trials, on a 20 m smooth walkway in the court which are separated by 2 10 min periods.	Pedar-X in shoe pedabarograph system Foot mask Insole A pair of standard canvas trainers	NM	Insole foot mask	BL mean peak plantar pressure Contact duration Contact area COP Bilateral average contact time	Peak pressure with LLD decreased at the lateral heel and medial heel. Pressure increased at the medial forefoot. Plantar region LLD increased at contact duration. Contact area decreased midfoot LLD. Locus COP increased during heel strike. There are significant differences in the plantar pressure distribution with LLD patients.
Ali et al. [2]	Stand still with feet a shoulder width apart. Data captured at rest. Then, subjects need to move one foot at a time into full pronation posture and full supination. Repeat the process for CL foot. Data is collected during the period of specified time of movement.	3D CODA MPX 30 motion analysis system Reflective markers	NM	NM	Foot pronation Foot supination	Experiment group: Changes in foot position from maximum pronation to supination in a limb length change of 1 cm
Krawiec et al. [21]	Take the measurement placements of leg length and innominate position while standing. Assistants read and record the inclinometer in degrees.	Palpation meter (PALM) inclinometer Caliper instrument Bubble inclinometer Tape measure Large paper clip	NM	NM	Degree of innominate position asymmetry	42 subjects (95%) had some degree of innominate position asymmetry. 32 subjects (73%) had right innominate rotated position. 2 subjects having no significant difference between the sagittal plane rotations of the right and left innominate.
O'Toole et al. [13]	Walk on the walkway for several times to familiarize with the surface and surrounding, about 20 m-long and 1.5 m-wide footplate levels Recorded with barefoot without LLD (control) 3 recording processes and repeated with 1 cm to 5 cm increment each	Polyurethane sole Sandal Musgrave footprint computerized pedabarograph system	56 Hz	Sandal with flexible polyurethane sole	Max load pressure Load distribution	LLD increased total loading on the short leg. LLD increased. Gait cycle times also changed. LLD increased the contact phase time.
Lopes et al. [22]	Stand still on a static position with eyes focused on the target located 1.5 m away for 30 s.	Berg balance scale (BSS) Photogrammetry (postural assessment software) Passive markers Cameras Force platform system (AMTI)	NM	NM	COP with Stabilometry: (i) ML standard and range (ii) AP standard and range (iii) Length (iv) Rectangular area (v) Elliptical area (vi) Average velocity (vii) Max. ML velocity (viii) Max. AP velocity	BSS shows no significant difference between the control and acromegaly groups. No significant difference for AP view between the control and acromegaly groups Right and left lateral view acromegaly group has greater significant difference than the control group. Stabilometry variables show the largest imbalance when the feet are together with eyes closed. Postural imbalance emphasizes in the acromegaly group.
Schneider et al. [23]	Stand in prone position. Clinician will do prone leg analysis, and results will recorded by a principal investigator. Repeat the process with another clinician.	Mechanical electric elevation treatment table	NM	Sole	NA	Change in the short leg with head rotation to left Change in the short leg with head rotation to right Change in the short leg with knees flexed observed on short leg Rotation of the head during prone leg analysis Derifield test appears to be unreliable. No significant correlation between the short leg and the patient-reported lower back pain There is significant difference noted in postural improvement
D'Amico et al. [24]	Stand still posture with kinematics recording based on an optoelectronic system	Stereophotogrammetric recording system Baropodographic platform	30 Hz	Wedges	Pelvic obliquity, averaged spinal offset, averaged global offset, Cobb's angle of main spine curve, lumbar lordotic angle, thoracic kyphosis angle, lower limb load balancing	

GRF: ground reaction force; ROM: range of motion; COM: center of mass; WD: weight distribution; LL: long limb; OS: offset; THA: total hip arthroplasty; LLD: leg length discrepancy; ML: medial-lateral; AP: anterior-posterior; OA: osteoarthritis: SD: standard deviation: COP: center of pressure: COF: center of occlusal force; LWD: lateral weight distribution; AWD: anterior weight distribution; Max.: maximum; NM: not mentioned; NA: not applicable; TPA: thermoplastic copolyamides.

**Table 4:** Summary of the kinematics and kinetics parameters related to LLD studies.

Author	Category	Plane	LL difference	Parameters		LLD	Control	*P* value
				Kinematics	Kinetics			
Swaminathan et al. [14]	Lowered leg	—	3.5 cm		WD	—	—	*P* < 0.05
	Raised leg (control)							*P* > 0.05
Zhang et al. [11]	Gait walker (LLD), shoe (control)	Coronal	4.6 cm	Ankle eversion (°)		−2.8 ± 4.6	−4.5 ± 2.3	*P* > 0.05
				Ankle ROM (°)		1.8 ± 4.9	8.7 ± 3.3	*P* < 0.05
				Hip adduction (°)		5.1 ± 3.6	5.8 ± 2.8	*P* > 0.05
				Hip ROM (°)		6.1 ± 2.0	8.3 ± 2.4	*P* < 0.05
				Knee adduction (°)		3.9 ± 2.2	4.6 ± 2.9	*P* > 0.05
				Knee ROM (°)		2.4 ± 2.5	3.3 ± 1.8	*P* > 0.05
		Sagittal		Ankle dorsiflexion (°)		11.1 ± 4.3	11.9 ± 3.4	*P* > 0.05
				Ankle ROM (°)		7.3 ± 4.3	5.7 ± 4.6	*P* > 0.05
				Hip extension (°)		2.4 ± 9.5	0.6 ± 10.9	*P* > 0.05
				Hip ROM (°)		37.2 ± 4.4	37.1 ± 5.4	*P* > 0.05
				Knee flexion (°)		22.7 ± 4.9	15.5 ± 8.7	*P* < 0.05
				Knee ROM (°)		9.5 ± 4.7	8.5 ± 5.4	*P* > 0.05
Resende et al. [3]	Short leg (control)	Coronal	1.45 cm	Hip adduction (°)		−6.89 ± 17.8	−1.82 ± 18.8	*P* > 0.001
				Pelvic obliquity decrease (°)		7.13 ± 11.2	0.23 ± 12.5	*P* < 0.001
					Ankle inversion moment (Nm·kg^−1^)	−0.09 ± 0.8	0.008 ± 0.8	*P* > 0.001
				Rearfoot inversion (°)		7.79 ± 21.0	1.65 ± 21.1	*P* > 0.001
				Rearfoot dorsiflexion (°)				*P* < 0.001
		Sagittal		Knee flexion (°)		16.2 ± 18.9	−5.4 ± 16.9	*P* > 0.001
				Hip flexion (°)		−10.08 ± 52.7	−2.44 ± 48.1	*P* < 0.001
					Ankle dorsiflexion moment (Nm·kg^−1^)	−18.03 ± 34.9	2.79 ± 35.2	*P* > 0.001
					Knee flexion moment (Nm·kg^−1^)	1.01 ± 0.9	−0.25 ± 1.0	*P* < 0.001
					Hip flexion moment (Nm·kg^−1^)	0.46 ± 1.56	0.05 ± 1.5	*P* < 0.01
	Long leg (control)	Coronal	1.45 cm	Rearfoot inversion (°)		0.36 ± 0.93	−0.22 ± 0.9	*P* < 0.01
				Hip adduction (°)		−9.44 ± 20.0	1.65 ± 21.1	*P* < 0.01
					Knee adduction	8.71 ± 23.3	−1.82 ± 18.8	*P* < 0.05
		Sagittal		Rearfoot dorsiflexion (°)	moment (Nm·kg^−1^)	−0.13 ± 0.7	0.03 ± 0.8	*P* < 0.05
				Knee flexion (°)		−10.8 ± 19.3	−5.4 ± 16.9	*P* < 0.05
				Hip flexion (°)		12.52 ± 53.9	−2.44 ± 48.1	*P* < 0.05
				Pelvic obliquity down (°)		15.24 ± 33.9	2.79 ± 35.2	*P* < 0.05
					Ankle dorsiflexion moment (Nm·kg^−1^)	−7.36 ± 14.7	0.23 ± 12.5	*P* < 0.001
						−0.77 ± 0.8	−0.25 ± 1.0	*P* < 0.001
					Ankle flexion moment (Nm·kg^−1^)	−0.5 ± 1.54	0.05 ± 1.5	*P* < 0.05
Walsh et al. [4]	LLDs, short leg	Coronal	0 cm	Pelvic obliquity (°)				
		Sagittal,	1 cm			—	0.3 ± 2.2	—
		Lateral	2 cm			2 cm	—	—
			2.5 cm			2.5 ± 1.4	—	—
			3 cm			3.4 ± 1.6	—	—
			5 cm			2.7 ± 1.7	—	—
			0 cm	Hip flexion (°)		2.7 ± 1.7	—	—
			1 cm			—	32.2 ± 3.6	—
			2 cm			33.5 ± 3.2	—	—
			2.5 cm			35.0 ± 3.0	—	—
			3 cm			35.1 ± 3.1	—	—
			5 cm			36.0 ± 3.8	—	—
			0 cm	Knee flexion (°)		39.7 ± 4.5	—	—
			1 cm			—	13.6 ± 5.4	—
			2 cm			16.9 ± 4.5	—	—
			2.5 cm			15.4 ± 3.6	—	—
			3 cm			14.5 ± 5.6	—	—
			5 cm			15.9 ± 2.7	—	—
			0 cm	Ankle flexion (°)		20.8 ± 4.5	—	—
			1 cm			—	53.3 ± 3.0	—
			2 cm			9.6 ± 3.0	—	—
			2.5 cm			11.2 ± 2.2	—	—
			3 cm			14.6 ± 3.6	—	—
			5 cm			15.1 ± 5.2	—	—
	LLDs, short leg	Coronal	0 cm	Knee flexion (°)		14.7 ± 5.0	—	—
		Sagittal	1 cm			—	49.8 ± 11.3	—
		Lateral	2 cm			48.2 ± 8.7	—	—
			2.5 cm			48.0 ± 7.1	—	—
			3 cm			48.3 ± 7.4	—	—
			5 cm			48.4 ± 7.0	—	—
			0 cm	Ankle flexion (°)		43.7 ± 7.0	—	—
			1 cm			—	−17.8 ± 6.9	—
			2 cm			−21.5 ± 5.0	—	—
			2.5 cm			−21.9 ± 5.8	—	—
			3 cm			−21.3 ± 6.3	—	—
			5 cm			−21.6 ± 4.9	—	—
						−24.3 ± 9.7	—	—
Resende et al. [18]	LLD, short leg	Sagittal	1.45 cm	Rearfoot plantar flexion (°)		—	—	*P* < 0.05
				Knee flexion (°)		—	—	*P* < 0.05
				Hip abduction (°)		—	—	*P* < 0.05
				Pelvic external-internal rotation (°)		—	—	*P* < 0.05
				Trunk external-internal rotation (°)		—	—	*P* < 0.05
					Ankle dorsiflexion moment (Nm·kg^−1^)	—	—	*P* < 0.05
					Knee flexion moment (Nm·kg^−1^)	—	—	*P* < 0.05
					Knee abduction moment (Nm·kg^−1^)	—	—	*P* < 0.05
					Hip flexion moment (Nm·kg^−1^)	—	—	*P* < 0.05
					Hip abduction moment (Nm·kg^−1^)	—	—	*P* < 0.05
Seeley et al. [17]	LLDs, long leg	Sagittal	<1 cm					
	LLDs, short leg			Hip (°)		0.99 ± 0.01	—	*P* > 0.05
				Knee (°)		0.99 ± 0.01	—	*P* > 0.05
				Ankle (°)		0.97 ± 0.02	—	*P* > 0.05
					Hip moment (Nm·kg^−1^)	0.94 ± 0.06	—	*P* > 0.05
					Knee moment (Nm·kg^−1^)	0.85 ± 0.11	—	*P* < 0.05
					Ankle moment (Nm·kg^−1^)	0.98 ± 0.02	—	*P* < 0.05
					Hip power (W·kg^−1^)	0.81 ± 0.14	—	*P* > 0.05
					Knee power (W·kg^−1^)	0.85 ± 0.07	—	*P* < 0.05
					Ankle power (W·kg^−1^)	0.94 ± 0.04	—	*P* < 0.05
			≥1 cm	Hip (°)		0.98 ± 0.04	—	*P* > 0.05
				Knee (°)		0.96 ± 0.08	—	*P* > 0.05
				Ankle (°)		0.87 ± 0.22	—	*P* > 0.05
					Hip moment (Nm·kg^−1^)	0.66 ± 0.37	—	*P* > 0.05
					Knee moment (Nm·kg^−1^)	0.96 ± 0.08	—	*P* < 0.05
					Ankle moment (Nm·kg^−1^)	0.86 ± 0.15	—	*P* < 0.05
					Hip power (W·kg^−1^)	0.41 ± 0.52	—	*P* > 0.05
					Knee power (W·kg^−1^)	0.50 ± 0.38	—	*P* < 0.05
					Ankle power (W·kg^−1^)	0.69 ± 0.31	—	*P* < 0.05
Wünnemann et al. [8]	Left leg, right leg (control)	Coronal	—			3.06	8.79	*P* < 0.05
				Hip adduction (°)		−11.66	−5.22	*P* < 0.05
				Hip abduction (°)		32.17	35.92	*P* < 0.05
				Hip flexion (°)		−15.24	−10.14	*P* < 0.05
				Hip extension (°)		0.84	0.94	*P* > 0.05
				Trunk forward tilt(°)		3.09	7.24	*P* < 0.05
				Pelvis lateral tilt (°)				
		Sagittal		Knee flexion (°)		0.55	0.65	*P* < 0.05
				Foot dorsiflexion (°)		1.24	1.11	*P* > 0.05
				Foot plantar flexion (°)		3.12	5.58	*P* < 0.05
				Step length (cm)		2.75	7.76	*P* < 0.05
				Single support time (s)		−14.88	−0.47	*P* < 0.05
					Hip extension moment (Nm·kg^−1^)	71.11	77.34	*P* > 0.05
					VGRF (N)	37.37	38.27	*P* > 0.05
					Knee flexion moment (Nm·kg^−1^)	−0.31	−0.05	*P* > 0.05
					Knee abduction moment (Nm·kg^−1^)	0.64	0.47	*P* > 0.05
					Ankle plantar flexion moment (Nm·kg^−1^)	1.62	1.01	*P* < 0.05
Park et al. [5]	Left leg, right leg (control)	—	0 cm	Step length (cm)		64.00 ± 6.15	63.29 ± 6.36	*P* > 0.05
			1 cm			64.71 ± 6.65	61.82 ± 6.26	*P* > 0.05
			2 cm			65.71 ± 7.35	62.35 ± 6.74	*P* < 0.05
			3 cm			66.35 ± 8.76	62.41 ± 7.38	*P* < 0.05
			0 cm	Step time (s)		0.63 ± 0.055	0.62 ± 0.06	*P* < 0.05
			1 cm			0.62 ± 0.064	0.63 ± 0.06	*P* < 0.05
			2 cm			0.61 ± 0.048	0.65 ± 0.06	*P* < 0.05
			3 cm			0.61 ± 0.046	0.66 ± 0.06	*P* < 0.05
			0 cm	Stride length (cm)		127.47 ± 12.6	—	*P* > 0.05
			1 cm			126.59 ± 12.8	—	*P* > 0.05
			2 cm			128.18 ± 14.1	—	*P* > 0.05
			3 cm			129.00 ± 15.9	—	*P* > 0.05
			0 cm	Stride time (s)		1.25 ± 0.11	—	*P* > 0.05
			1 cm			1.25 ± 0.13	—	*P* > 0.05
			2 cm			1.26 ± 0.10	—	*P* > 0.05
			3 cm			1.26 ± 0.10	—	*P* > 0.05
			0 cm	Single leg support (%)		34.13 ± 1.51	34.68 ± 1.04	*P* < 0.05
			1 cm			34.49 ± 1.51	34.21 ± 1.11	*P* < 0.05
			2 cm			35.41 ± 1.60	33.92 ± 1.11	*P* < 0.05
			3 cm			35.78 ± 1.75	33.39 ± 1.27	*P* < 0.05
			0 cm	Double leg support (%)		31.21 ± 2.22	—	*P* > 0.05
			1 cm			31.30 ± 2.07	—	*P* > 0.05
			2 cm			30.67 ± 2.13	—	*P* > 0.05
			3 cm			30.84 ± 2.26	—	*P* > 0.05
Stief et al. [16]	LCPDs, short leg (control)	Coronal	1.10 ± 0.53		Max. adduction knee moment (Nm·kg^−1^)	0.14 ± 0.11	0.26 ± 0.08	*P* ≤ 0.05
					Max. adduction hip moment (Nm·kg^−1^)	0.42 ± 0.04	0.58 ± 0.11	*P* ≤ 0.05
		Sagittal		Max. knee flexion (°)		19.5 ± 9.0	22.8 ± 6.1	*P* ≤ 0.05
				Max. knee extension (°)		10.9 ± 5.8	5.9 ± 3.3	*P* ≤ 0.05
				Knee ROM (°)		11.8 ± 4.1	17.3 ± 6.0	*P* ≤ 0.0
				Max. hip flexion (°)		29.9 ± 7.6	35.7 ± 5.3	*P* ≤ 0.05
				Max. hip extension (°)		−3.0 ± 9.6	−10.3 ± 4.9	*P* ≤ 0.05
				Hip ROM (°)		33.2 ± 9.8	46.7 ± 6.0	*P* ≤ 0.05
				Max. pelvis obliquity (°)		3.7 ± 3.3	4.4 ± 2.9	*P* ≤ 0.05
				Pelvis obliquity ROM (°)		7.7 ± 2.7	9.9 ± 3.8	*P* ≤ 0.05
Maeda et al. [9]	Right leg	Coronal	0.1 cm		WD (%)	—	—	*P* > 0.05
	Left leg (control)	Sagittal	≥0.6 cm		WD (%)	—	—	*P* < 0.05
	Left leg	Coronal	0.1 cm		WD (%)	—	—	*P* > 0.05
	Right leg (control)	Sagittal	≥0.4 cm		WD (%)	—	—	*P* < 0.05
Faraj et al. [10]	LLD, right leg, short left leg (control)	Coronal	±2.25 cm	Mean peak plantar pressure (kpa)		400	300	
								—
				Stance contact duration (msec)		750	500	
						45	35	—
				Contact area (cm2)				—
Ali et al. [2]	LLD, right leg	Sagittal	1 cm	Max. foot pronation		—	—	*P* < 0.05
	Short left leg (control)			Max. foot supination		—	—	*P* > 0.05
Lopes et al. [22]	LLD, short leg (control)	Coronal	—	HHA (°)		1.02 ± 3.86	0.68 ± 3.57	*P* > 0.05
				AHA (°)		−0.07 ± 2.70	0.31 ± 1.67	*P* > 0.05
				ASISHA (°)		0.07 ± 2.70	−0.23 ± 2.01	*P* > 0.0
				AcASHA (°)		0.14 ± 3.35	−0.52 ± 2.67	*P* > 0.05
				RLFA (°)		−2.98 ± 5.65	−2.59 ± 4.76	*P* > 0.05
				LLFA (°)		−2.70 ± 4.01	−3.19 ± 5.12	*P* > 0.05
				DBLL (°)		−0.74 ± 5.66	1.38 ± 5.09	*P* > 0.05
				TTHA (°)		0.75 ± 2.27	1.31 ± 1.71	*P* > 0.05
				RHA (°)		17.3 ± 13.1	23 ± 15.9	*P* > 0.05
				LHA (°)		19.8 ± 16	20.4 ± 12.4	*P* > 0.05
				ST3Has (cm)		0.08 ± 24.8	1.12 ± 24.8	*P* > 0.05
				RLHA (°)		2.30 ± 8.97	6.58 ± 8.21	*P* > 0.05
				LLHA (°)		5.60 ± 8.79	5.98 ± 8.80	*P* > 0.05
		Sagittal	—	HHA (R) (°)		39.6 ± 7.81	40.7 ± 8.50	—
				HVA (R) (°)		23.5 ± 11.5	20.8 ± 12.7	—
				TVA (R) (°)		−5.88 ± 3.50	−2.85 ± 3.22	—
				HA (R) (°)		−12.7 ± 7.55	−6.63 ± 5.53	—
				BVA (R) (°)		1.25 ± 1.50	1.37 ± 1.39	—
				PHA (R) (°)		−12.7 ± 6.96	−18.1 ± 9.57	—
				KA (R) (°)		−1.62 ± 7.35	1.18 ± 6.23	—
				AA (R) (°)		84.8 ± 3.90	85.1 ± 3.40	—
				HHA (L) (°)		39.1 ± 7.91	41.6 ± 9.20	—
				HVA (L) (°)		24 ± 10.2	19.5 ± 12.3	—
				TVA (L) (°)		−5.31 ± 3.40	−3.23 ± 3.17	—
				AHA (°)		−0.07 ± 2.70	0.31 ± 1.67	*P* > 0.05
				HA (L) (°)		−12.5 ± 6.88	−8.31 ± 5.41	—
				BVA (L) (°)		1.36 ± 1.71	1.71 ± 1.24	—
				PHA (L) (°)		−12.3 ± 9.02	−20.2 ± 6.20	—
				KA (L) (°)		−2.71 ± 6.85	−0.68 ± 5.19	—
				AA (L) (°)		85.5 ± 3.98	85.4 ± 3.23	—

HHA: head horizontal alignment; AHA: acromion horizontal alignment; ASISHA: anterior-superior iliac spine horizontal alignment; AcaSISA: angle between the acromion and anterior-superior iliac spine alignment; RLFA: right limb frontal angle; LLFA: left limb frontal angle; DBLL: difference between lower limbs; TTHA: tibia tuberosity horizontal angle; RHA: right hip angle; LHA: left hip angle; RLHA: right leg-heel angle; LLHA: left leg-heel angle; HVA: head vertical alignment; TVA: trunk vertical alignment; HA: hip angle; BVA: body vertical alignment; PHA: pelvis horizontal alignment; KA: knee angle; AA: ankle angle; BL: bilateral; FP: foot pronation; FS: foot supination; LWD: lateral weight distribution; AWD: anterior weight distribution; BS: bilateral-sagittal; CL: contralateral; (R): right side; (L): left side; (°): angle

**Table 5:** Summary of the parameters related to postural balance.

Author	Category	Sample rate (Hz/second)	LL difference	Parameter	LLD	Control	*P* value
Zhang et al. [11]	Foot with equalizer walker	600 Hz	4.6 cm	COM (m)	—	—	*P* < 0.05
					LL	SL	
Mahar et al. [6]	Long leg, short leg (control)	100 Hz with 20 s	0 cm	Mean COP (mm)	47.4 ± 5.3	47.4 ± 5.3	^∗^ *P* > 0.05^LL^, *P* > 0.05^RL^
			1 cm	Mean COP (mm)	42.5 ± 4.8	54.7 ± 5.1	*P* < 0.05^LL^, *P* > 0.05^RL^
			2 cm	Mean COP (mm)	42.9 ± 7.5	56.9 ± 5.0	*P* < 0.051^LL^, *P* < 0.05^RL^
			3 cm	Mean COP (mm)	40.5 ± 6.6	52.1 ± 10.6	^∗^ *P* > 0.05^LL^, *P* < 0.05^RL^
			4 cm	Mean COP (mm)	40.5 ± 8.8	55.7 ± 10.7	*P* < 0.05^LL^, *P* < 0.05^RL^
Murrell et al. [19]	LLD (control)	10 Hz with 12.8 s	0.95 cm	COP (AP with EO) (mm)	47.7 ± 14.5	39.4 ± 6.0	*P* > 0.05
				COP (AP with EC) (mm)	58.8 ± 19.0	54.8 ± 13.5	*P* < 0.05
				COP (ML with EO) (mm)	27.7 ± 9.0	27.2 ± 6.6	*P* > 0.05
				COP (ML with EC) (mm)	40.8 ± 15.2	40.1 ± 9.9	*P* < 0.05
			0 cm	COP path length (mm)	49.81 ± 21.70	—	*P* < 0.05
Park et al. [5]	Left leg, right leg (control)	—	1 cm	COP path length (mm)	65.71 ± 32.68	—	*P* < 0.05
			2 cm	COP path length (mm)	60.38 ± 22.93	—	*P* < 0.05
			3 cm	COP path length (mm)	83.16 ± 34.05	—	*P* < 0.05
						—	
Maeda et al. [9]	Right leg, left leg (control)	—	0.1 cm	COP trajectory length, area (mm^2^/s)	—	—	*P* > 0.05
	Left leg, right leg (control)		≥0.6cm	COP trajectory length, area (mm^2^/s)	—		*P* < 0.05
			0.1 cm	COP trajectory length, area (mm^2^/s)	—	—	*P* > 0.05
			≥0.4cm	COP trajectory length, area (mm^2^/s)	—	—	*P* < 0.05
Faraj et al. [10]	LLD, right leg, short left leg (control)	—	±2.25 cm		—		
						—	—
Lopes et al. [22]	LLD, short leg (control)	—	—	COP trajectory length (mm)		—	*P* < 0.05
				COP (AP with EO) (mm)	—	—	*P* < 0.05
				COP (AP with EC) (mm)	—	—	*P* < 0.05
				COP (ML with EO) (mm)	—	—	*P* < 0.05
				COP (ML with EC) (mm)	—		*P* < 0.05

LL: left leg; RL: right leg; COP: center of pressure; AP: anterior-posterior; ML: medial-lateral; EO: eyes open; EC: eyes closed.
